# Molecular assessment of *Chlamydia psittaci* and *Circovirus* in psittacines from a CETAS in Bahia, Brazil

**DOI:** 10.1007/s42770-025-01649-2

**Published:** 2025-02-26

**Authors:** Edma Santos Antonio, Ricardo Evangelista Fraga, Priscila Sacramento, Ayane Lima de Freitas, Ana Clara Barbosa Santana, Sabrina Barbosa, Ramon Costa Dominato, Janisete Gomes Silva

**Affiliations:** 1 Departamento de Ciências Biológicas, Universidade Estadual de Santa Cruz, Campus Soane Nazaré de Andrade, Ilhéus, BA 45662-900 Brazil; 2 Instituto Multidisciplinar em Saúde, Universidade Federal da Bahia, Campus Anísio Teixeira, Vitória da Conquista, Salvador, BA 45029-094 Brazil; 3 Universidade Estadual do Sudoeste da Bahia, Campus Jequié, Jequié, BA 45205-490 Brazil

**Keywords:** BFDV, Behavior, Chlamydiosis, Conservation, Quarantine, Release

## Abstract

Mistreatment and unsanitary conditions to which trafficked animals are subjected provide an environment conducive to the proliferation and dissemination of pathogens. The Centros de Triagem de Animais Silvestres (CETAS - Wild Animal Screening Centers), which receive trafficked animals, aim to release them back into the wild, thus making the investigation of pathogens essential. The objective of this study was to conduct an epidemiological study of infections by *Chlamydia psittaci* and *Circovirus* in psittacines from wildlife trafficking housed at a CETAS in Bahia. Cloacal swab and blood samples were collected from 135 psittacines, both residents and newly arrived in quarantine, housed at the CETAS in Vitória da Conquista, Bahia. The presence of pathogens was determined by conventional PCR. The PCRs consisted of amplifying the *opmA* gene and ORF1 to detect *C. psittaci* and *Circovirus*, respectively. For *C. psittaci*, three (2.2%) animals were diagnosed as positive, then treated with antibiotics, retested, and included in the CETAS population after testing negative for the bacterium. Among the 135 psittacines evaluated, 22 (16%) showed feathering abnormalities despite testing negative for *Circovirus*. This research was the first epidemiological survey of *Circovirus* infection in psittacines in Bahia and improved the sanitary safety of wildlife release programs.

## Introduction

The mortality rate among trafficked animals is high due to mistreatment and poor sanitary conditions [1]. Such conditions also create an environment conducive to the proliferation and spread of pathogens [2] such as *Chlamydia psittaci* [3] and *Circovirus* [4].

The bacterium *C. psittaci* is one of the most common psittacine pathogens, which cause several outbreaks of infections documented worldwide, including in Brazil. The serotype or genotype A is most commonly associated with this group of birds [5–7]. Chlamydiosis has a wide clinical spectrum ranging from mild clinical signs to systemic infection, which is due to its ability to infect various organs in the cardiovascular, digestive, lymphatic, neurological, reproductive, respiratory, and urinary systems, potentially leading to death. However, there are no pathognomonic clinical signs. The variation in symptoms may be related to several factors such as animal species, age, level of immune compromise, and bacterial serotype [8].

Avian *Circovirus*, also called the BFDV - Beak and Feather Disease Virus, causes the well-known Psittacine Beak and Feather Disease (PBFD), a contagious disease with high morbidity and mortality, especially in chicks [9]. Key clinical signs of the disease include bends and fractures along the shaft of feathers, development of dysplastic feathers, retention and thickening of feather sheaths, hemorrhage in the feather follicle, feather loss or absence of feather growth, overgrowth or irregularity of the beak, longitudinal splits in the beak, and necrosis of the palatine mucosa [10]. In some species, the infection may be less fatal, but animals typically succumb to secondary infections resulting from immunosuppression caused by the virus [11].

In both infections aforementioned, asymptomatic carriers may exist [3, 12, 13]. For captive animals with potential for release, subclinical infection poses an environmental risk, potentially leading to outbreaks of diseases caused by these pathogens in wildlife. Thus, in order to mitigate the impacts of wildlife trafficking on fauna, Centros de Triagem de Animais Silvestres (CETAS - Wild Animal Screening Centers) were established in Brazil. These centers are responsible for receiving, evaluating, rehabilitating, and ideally releasing wildlife, primarily from enforcement actions against trafficked shipments [14, 15].

Outbreaks of chlamydiosis in psittacines have been documented in a CETAS in Bahia [3]. This disease is highly debilitating for infected birds and is also a zoonosis with human fatalities recorded [5, 16]. It is recommended that captive birds undergo periodic testing for the presence of the pathogen to prevent potential new outbreaks of the disease in both animals and humans [14]. Additionally, as a preventive measure, newly arrived psittacines at a CETAS should be quarantined for 30 days or until tested negative for *C. psittaci* before integration [8].

Technicians from a CETAS in Bahia have documented psittacines with abnormal plumage, which may be related to PBFD. Therefore, investigating the presence of *Circovirus* is of utmost importance, as it is a virus of significance for birds [14]. *Circovirus* has been extensively studied in countries where psittacines are found, whether native or introduced [17, 18], but few studies have been carried out on native brazilian psittacines [4, 19]. Brazil does not figure among the countries in the routes of psittacine virus transmission [20]; however, it is not yet fully understood whether Brazilian psittacines, or neotropical species in general, are less susceptible to the virus or if the virus distribution has not been adequately mapped in the Neotropical region.

The aim of this study was to carry out an epidemiological investigation of infections by *C. psittaci* and *Circovirus* in psittacines recovered from wildlife trafficking and housed at a CETAS in Bahia. Our specific objectives were as follows: (i) to catalog the species of Psittacidae received by the CETAS during the study period; (ii) to determine the sex ratio of the analyzed population; (iii) to measure the stress levels of the birds included in the study; (iv) to assess the feather score of the individuals; (v) to catalog clinical signs indicative of infections; (vi) to test whether there is a difference in stress levels between males and females and between psittacines with and without feather issues; (vii) to verify whether there is a correlation or association between the variables “feather score” and “stress index”; (viii) to report the frequency of animals infected with *C. psittaci* and/or *Circovirus* at the CETAS; and (ix) to observe whether there is a correlation between clinical signs (including feather issues) and infection by either of the two pathogens.

## Materials and methods

### Study site

Biological samples were collected from birds housed at the CETAS in Vitória da Conquista, Bahia, Brazil (CETAS-VDC). Clinical observations of the animals and biological samples were carried out at the CETAS. Sample processing was performed at the Laboratório de Biologia Celular e Molecular of the Instituto Multidisciplinar em Saúde (IMS/UFBA). This research project was approved by the Animal Ethics Committee of IMS/UFBA (protocol no. 093/2021).

All small and medium-sized psittacines housed at CETAS-VDC and birds that arrived from November 2022 to September 2023 were evaluated. Larger psittacines, such as macaws, were kept in a separate physical location and not all were evaluated due to handling difficulties. Newly arrived birds were quarantined and integrated into the CETAS population only after the results of their health assessments were released. The quarantine protocol at CETAS-VDC involves keeping new animals in cages within a closed room separate from the aviaries where resident animals are housed. During this period, animals undergo clinical evaluation, testing for detection of pathogens relevant to each species, and treatment for possible infections if necessary.

### Collection of biological material

A total of 135 adult individuals from 13 species of the Psittacidae family were analyzed. Cloacal swabs and blood were collected from all animals. Cloacal swab samples were used for investigating *C. psittaci* and *Circovirus*. Sterile swabs (Goodwood Medical Care^®^, Dalian, LN, China) used for collection were placed in 2 ml of ultrapure water in sterile tubes and kept refrigerated (4 °C) until processing (within 24 h post-collection) [3]. Blood, collected via nail clipping, was used for molecular sexing, leukocyte evaluation, and *Circovirus* detection. Blood for molecular analyses was spotted onto Qualy^®^ qualitative filter paper (JProlab, Paraná, Brazil) and stored at room temperature until processing.

### Blood smear and leukocyte evaluation

For each individual, a drop of blood obtained from a small nail clip was placed on a glass slide to create a blood smear. Using an Olympus^®^ CX31 Optical Microscope, total leukocyte count (indirect method) and leukocyte differential count were performed. The heterophil/lymphocyte (H: L) ratio was calculated as an index of stress [21].

### Clinical and plumage evaluation

During the collection of biological samples, each bird underwent clinical evaluation by a veterinarian from CETAS, following the guidelines of Caldas [22] and Grespan and Raso [23]. Clinical assessments included body condition score (1 to 5), presence of ocular or nasal discharge, normality of self-cleaning activity, consistency and color of feces (when observable), normality of beak and claw morphology and health, presence of ectoparasites, feather condition, and other behavioral aspects.

To quantify the degree of feather abnormalities as a possible clinical sign of PBFD, a plumage evaluation of the psittacines was conducted. For this analysis, photographic records were taken after physical restraint of the birds. The evaluation followed the ten-point scoring system proposed by Meehan et al. [24]. This system assesses feathers, plumes, and skin lesions observed in five different parts of the body: chest/flank, back, legs, wings, and tail. Each area was scored from 0 (severe feather compromise) to 2 (minimal feather compromise), and the sum of these subscores generated a final score ranging from 0 to 10, where 0 indicates the highest compromised feather score and 10 represents intact plumage.

### Molecular sexing

A 5 × 5 mm fragment of the filter paper containing the collected blood was cut and immersed in 100 µl of Digsol buffer (20 mM EDTA, 120 mM NaCl, 50 mM Tris-HCl). The tube was incubated in a dry block shaker at 55 °C for 3 h or at 37 °C overnight. After this period, the paper was discarded, and the liquid medium underwent DNA extraction using the Virus RNA + DNA Preparation Kit (Cellco Biotech^®^), following the manufacturer’s instructions. We added 250 µl of lysis buffer to the initial sample that was subsequently vortexed for 15 s, and kept at room temperature for 10 min. Then, 250 µl of absolute ethanol were added to the lysate, vortexed for 5 s, added to the silica column, and centrifuged at 13,000 g for 1 min. The column was washed with wash buffers A and B, and finally, DNA was recovered from the column by adding 40–50 µl of elution buffer and centrifuging at 13,000 g for 1 min.

The DNA extracted from blood was amplified using the Polymerase Chain Reaction (PCR) for molecular sexing with the primers 2550F (5’-GTTACTGATTCGTCTACGAGA-3’) and 2718R (5’-ATTGAAATGATCCAGTGCTTG-3’), which flank a region of the CHD-1 gene [25]. The standardized protocol by Antonio et al. [26] was used with the following adaptations: 1x buffer; 1.5 mM MgCl2; 0.4 mM dNTPs; 1 µM of each primer; 1.5 U of Taq DNA polymerase; 4 µl of extracted DNA, and ultrapure H2O to a total volume of 20 µl. The amplification was performed in a SimpliAmp™ Thermal Cycler (Applied Biosystems™) with the following thermocycling program: initial denaturation at 94ºC for 3 min; 40 cycles of (i) denaturation at 94ºC for 30 s, (ii) annealing at 50ºC for 30 s, and (iii) extension at 72ºC for 45 s; followed by a final extension at 72ºC for 45 s. PCR products were visualized on a 2% agarose gel stained with ethidium bromide. Sex identification is crucial as psittacines lack sexual dimorphism, and in this case, it also served as an internal control for extraction, as the same material was used for *Circovirus* detection.

### Detection of *Chlamydia psittaci*

The cloacal swab material underwent DNA extraction by boiling, following the method described by Fan et al. [27]. A volume of 1 ml of biological sample was centrifuged at 14,000 rpm for 15 min (Microcentrifuge Spinlab SL-5GR), the supernatant was discarded, and the pellet was resuspended in 100 µl of phosphate-buffered saline (PBS) (150 mM, pH 7.2), then centrifuged again at 14,000 rpm for 15 min (Microcentrifuge Spinlab SL-5GR). After two washes with PBS, the pellet was resuspended in 40 µl of PBS and heated at 100 °C for 10 min. The sample underwent thermal shock (-20 °C) and was then centrifuged at 14,000 rpm for 5 min (Microcentrifuge Spinlab SL-5GR). Finally, the supernatant containing DNA was stored at -20 °C until processing for the molecular test for *C. psittaci*. A positive control for *C. psittaci* (Felocell CVR-C^®^ vaccine, Zoetis, São Paulo, Brazil) was included both in DNA extraction and in all PCR series performed.

The DNA extracted from cloacal swabs with PBS was subjected to PCR optimized by Mirabal-Santos et al. [3], using the primers CPF (5’-GCAAGACACTCCTCAAAGCC-3’) and CPR (5’-CCTTCCCACATAGTGCCATC-3’) developed by Hewinson et al. [28] that amplify the ompA gene. The PCR reaction mixture consisted of 1x buffer (200 mM Tris-HCl pH 8.4, 500 mM KCl), 2 mM MgCl2, 0.08 mM dNTPs, 0.1 µM of each primer, 1.25 U Taq DNA polymerase, 5 µl of extracted DNA, and ultrapure H_2_O to a total volume of 50 µl. All PCR reagents were from Invitrogen™. The amplification was performed in a SimpliAmp™ Thermal Cycler (Applied Biosystems™) with the following thermocycling program: initial denaturation at 94ºC for 3 min; 40 cycles of (i) denaturation at 94ºC for 30 s, (ii) annealing at 50ºC for 30 s, and (iii) extension at 72ºC for 45 s; followed by a final extension at 72ºC for 45 s. PCR products were visualized on a 2% agarose gel stained with ethidium bromide, where the presence of a 264 bp amplicon indicated a positive result.

Individuals positive for chlamydiosis were treated with injectable enrofloxacin (1.5 mg/kg), administered intramuscularly once daily for 45 days, following the protocol described by Flammer [29] and Fowler and Cubas [30]. They were retested after completing the treatment regimen.

### Detection of *Circovirus*

The DNA extraction from the cloacal swab for *Circovirus* evaluation was carried out using the phenol/chloroform extraction method described by Green and Sambrook [31]. A 250 µl aliquot of the liquid in which the swab was soaked was transferred to a microcentrifuge tube, to which 10 µl of Proteinase K (10 mg/ml) was added. The tube was incubated in a dry block shaker at 55 °C for 3 h or overnight at 37 °C. Then, 250 µl of phenol/chloroform solution (1:1; pH 7.0) were added to the sample, the contents were emulsified, and centrifuged at 14,000 rpm for 1 min (Microcentrifuge Spinlab SL-5GR). The aqueous phase was transferred to a new microcentrifuge tube, to which an equal volume of chloroform was added. The mixture was emulsified and centrifuged at 14,000 rpm for 1 min (Microcentrifuge Spinlab SL-5GR). The aqueous phase was again collected and twice the volume of this phase was added to ice-cold 95% ethanol. The mixture was kept at -20 °C for 20 min and then centrifuged again at 14,000 rpm for 10 min (Microcentrifuge Spinlab SL-5GR) to pellet the DNA. The DNA pellet was air-dried with the tube open on a heating plate at 55 °C for 24 h. After ethanol drying, the DNA was resuspended in ultrapure H_2_O.

The positive control for *Circovirus*, liver and spleen material from infected animals, underwent both DNA extractions (phenol/chloroform and Cellco Biotech^®^ Kit) and was included in all PCR runs performed.

For the detection of *Circovirus*, DNA extracted from both samples (blood extracted with kit and swab extracted with phenol/chloroform) underwent conventional PCR using the primer pair P2 (5’-AACCCTACAGACGGCGAG-3’) and P4 (5’-GTCACAGTCCTCCTTGTACC-3’) developed by Ypelaar et al. [32], which amplify a fragment of the BFDV ORF V1. The reaction had a total volume of 25 µl, with 2 µl of template DNA (approximately 50 ng) and the remaining reagents at the following concentrations: 1X buffer (200 mM Tris-HCl pH 8.4, 500 mM KCl), 4 mM MgCl2, 0.15 mM dNTP, 0.8 µM of each primer, and 1 U Taq DNA polymerase. All PCR reagents were from Invitrogen™. The thermal cycling conditions were as follows: initial denaturation at 94 °C for 2 min; followed by 32 cycles of 94 °C for 1 min, 60 °C for 30 s, and 72 °C for 1 min; with a final extension at 72 °C for 7 min [12]. PCR products were visualized on a 2% agarose gel stained with ethidium bromide where the presence of a 717 bp amplicon indicated a positive result.

### Statistical analysis

The Shapiro-Wilk test was used to assess the normal distribution of the variables. The Wilcoxon test for non-parametric data was employed to compare the stress levels between the group with feather problems and the group without feather problems. The same test was used to compare stress levels between males and females. To examine the potential relationship or correlation between the variables “feather score” and “stress index,” the following tests were applied to the data from birds with feather problems: Linear regression, Spearman correlation, and Kendall correlation. Principal Component Analysis (PCA) was carried out to describe the relationships between the “feather score” and “stress index” (H: L) variables. Results were obtained using the factoextra 1.0.7 package [33] and PCA plots were generated using the ggplot2 package. Statistical significance was considered for p-values ≤ 0.05, outliers were removed, and all analyses were performed using R version 4.2.0 [34].

## Results

### Species evaluated

Among the 13 species of Psittacidae studied in the present work, the most representative were *A. aestiva* (blue-fronted parrot) (54/135; 40%) and *Eupsittula cactorum* (cactus parakeet) (31/135; 23%). Molecular sexing was successful, thus demonstrating the integrity of the extracted DNA and revealing a sex ratio of 1:1.6 (female: male) (Table 1).

### Stress index

**Fig. 1:**
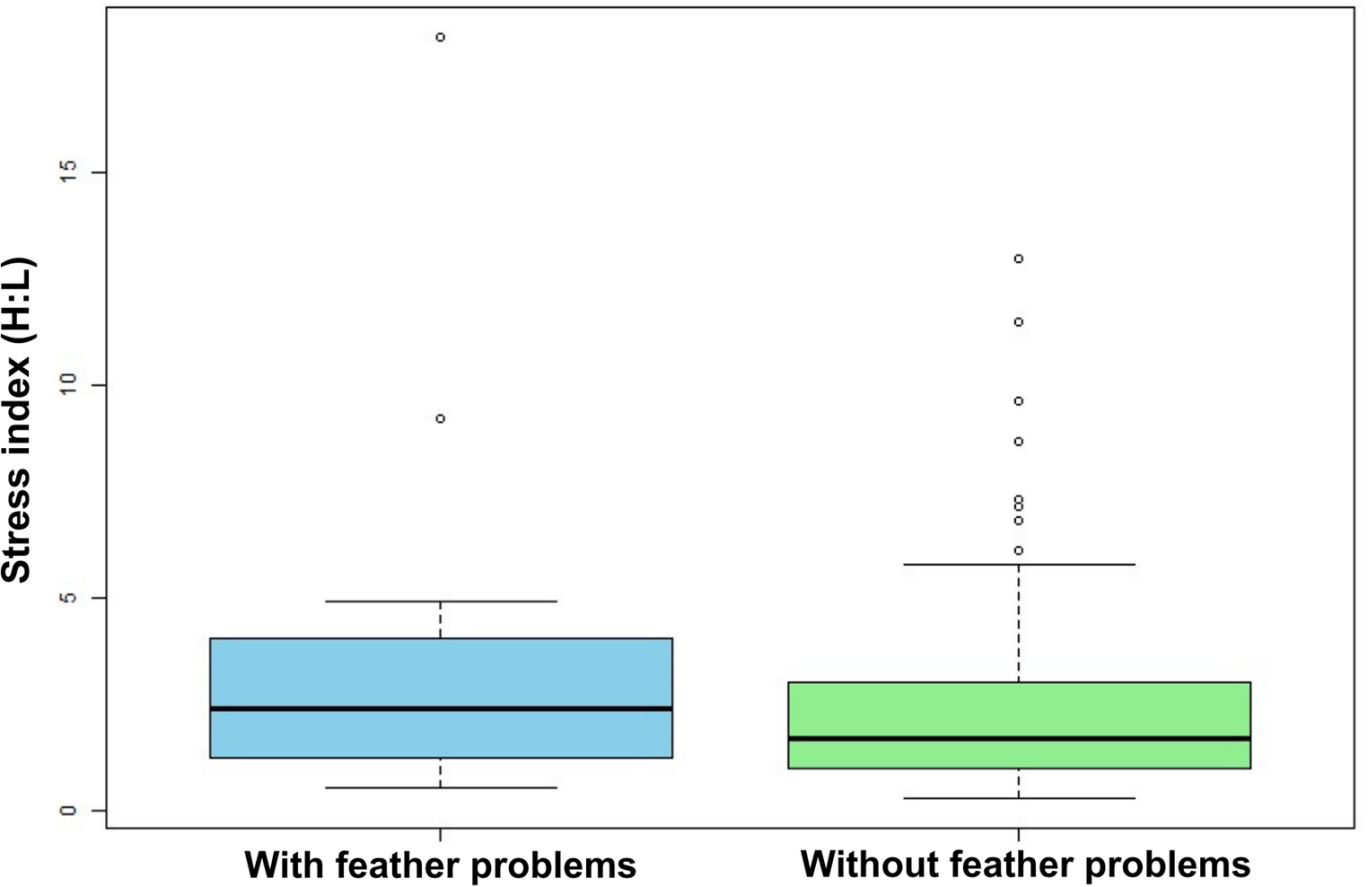
Mean and standard deviation of the stress index for animals with and without feather problems

The heterophil/lymphocyte ratio (H: L) ranged from 0.54 to 18.2 among birds with feather problems and/or other clinical signs of PBFD, with a general mean of 3.6 ± 4.0 (Table 2), whereas other birds had a mean of 2.8 ± 3.6. The expected value for non-stressed psittacines does not exceed 0.7 [35, 36]. The data obtained did not follow a normal distribution (p-value = 2.512e-06 for birds with feather problems; p-value = 2.834e-12 for healthy birds). Consequently, the Wilcoxon test was applied, resulting in a p-value of 0.2233 (> 0.05). Thus, no statistically significant difference in stress index was observed between the groups with and without feather problems (Fig. 1).

Overall, males showed a higher mean stress index than females (Table 1). Despite this, when comparing the stress index between males and females using the Wilcoxon test, a p-value of 0.0876 was obtained, indicating that there was no statistically significant difference.

**Table 1:** Psittacines evaluated and results of the analyses

Scientific name	Common name	Molecular sexing		Heterophil/lymphocyte ratio		Body condition score	Feather problems	*Chlamydia psittaci* positives	*Circovirus* positives
		Male	Female	Male	Female				
*Amazona aestiva*	Turquoise-fronted Parrot	37/54	17/54	2.1 ± 1.7	1.5 ± 1.1	2.9 ± 0.2	5/54	0/54	0/54
*Eupsittula cactorum*	Cactus Parakeet	23/31	8/31	4.6 ± 4.1	4.9 ± 4.9	2.9 ± 0.2	6/31	0/31	0/31
*Ara ararauna*	Blue-and-yellow Macaw	4/8	4/8	2.4 ± 1.0	4.1 ± 3.6	3	2/8	2/8	0/8
*Primolius maracana*	Blue-winged Macaw	3/6	3/6	2.6 ± 0.6	1.3 ± 0.5	3	0/6	0/6	0/6
*Psittacara leucophthalmus*	White-eyed Parakeet	4/6	2/6	11.1 ± 13.7	2.0 ± 0.5	3	3/6	0/6	0/6
*Aratinga auricapillus*	Golden-capped Parakeet	1/5	4/5	1.6	2.9 ± 3.9	3	2/5	0/5	0/5
*Brotogeris chiriri*	Yellow-chevroned Parakeet	1/5	4/5	0.4	2.2 ± 3.1	3	2/5	0/5	0/5
*Eupsittula aurea*	Peach-fronted Parakeet	4/5	1/5	2.5 ± 1.9	0.5	3	0/5	0/5	0/5
*Amazona rhodocorytha*	Red-browed Parrot	1/4	3/4	0.8	1.7 ± 1.4	3	0/4	0/4	0/4
*Thectocercus acuticaudatus*	Blue-crowned Parakeet	4/4	0/4	1.8 ± 0.8	-	3	1/4	1/4	0/4
*Alipiopsitta xanthops*	Yellow-faced Parrot	1/3	2/3	2.0	2.1 ± 0.4	3	0/3	0/3	0/3
*Orthopsittaca manilatus*	Red-bellied Macaw	0/2	2/2	-	2.7 ± 2.3	3	1/2	0/2	0/2
*Amazona amazonica*	Orange-winged Parrot	1/2	1/2	1.6	0.8	3	0/2	0/2	0/2
**Total**		**84/135**	**51/135**	**3**.**2 ± 4**.**2**	**2**.**5 ± 2**.**8**	**3**.**0 ± 0**.**2**	**22/135**	**3/135**	**0/135**

**Table 2:** Psittacines with feather problems

Individual	Species	Sex	Heterophil/ lymphocyte ratio	Evaluation of ten-point scores^a^						Other clinical signs
				Chest/flank	Back	Legs	Wings	Tail	Total score	
1	*Psittacara leucophthalmus*	Male	2.93	0	0.25	0	0.5	0	0.75	-
2	*Thectocercus acuticaudatus*	Male	2.75	0.25	0.25	0	1.5	2	4	-
3	*Ara ararauna*	Female	9.22	1.25	1.5	1.25	1	0	5	-
4	*Amazona aestiva*	Female	0.70	0.75	0.75	0.75	1	2	5.25	-
5	*Psittacara leucophthalmus*	Female	2.38	0.25	2	0.25	2	1	5.5	Overgrown beak
6	*Ara ararauna*	Male	3.16	1.5	1.25	1.5	1.5	1	6.75	-
7	*Psittacara leucophthalmus*	Male	10.25	1.5	2	2	2	0	7.5	-
8	*Amazona aestiva*	Male	0.85	2	2	2	1.5	0	7.5	Feathers around the cloaca dirty and body condition score 2
9	*Orthopsittaca manilatus*	Female	4.35	2	2	2	2	0	8	-
10	*Amazona aestiva*	Male	3.75	1.5	2	1.5	2	1	8	Lack of self-cleaning
11	*Brotogeris chiriri*	Female	0.54	2	2	2	2	0	8	-
12	*Eupsittula cactorum*	Male	18.20	2	2	2	2	0	8	-
13	*Eupsittula cactorum*	Female	2.69	2	2	2	2	0	8	-
14	*Eupsittula cactorum*	Male	2.19	2	2	2	2	0	8	-
15	*Eupsittula cactorum*	Male	1.42	2	2	2	2	0	8	-
16	*Brotogeris chiriri*	Female	1.17	2	2	2	2	0	8	-
17	*Eupsittula cactorum*	Male	1.02	2	2	2	2	0	8	-
18	*Amazona aestiva*	Male	4.94	2	2	2	0	2	8	Self-mutilation on the wing and overgrown beak
19	*Aratinga auricapillus*	Female	1.33	0.75	2	1.5	2	2	8.25	-
20	*Eupsittula cactorum*	Male	4.33	2	0.25	2	2	2	8.25	-
21	*Aratinga auricapillus*	Male	1.56	1.5	2	2	2	1	8.5	-
22	*Amazona aestiva*	Male	0.67	2	2	2	2	1	9	-
23	*Amazona aestiva*	Male	4.47	2	2	2	2	2	10	Overgrown beak and dirty cloacal feathers
24	*Amazona aestiva*	Male	1.50	2	2	2	2	2	10	Overgrown beak
**Mean**			3.6 ± 4.0							

^a^Meehan et al. [24]

### Clinical and plumage assessment

**Fig. 2:**
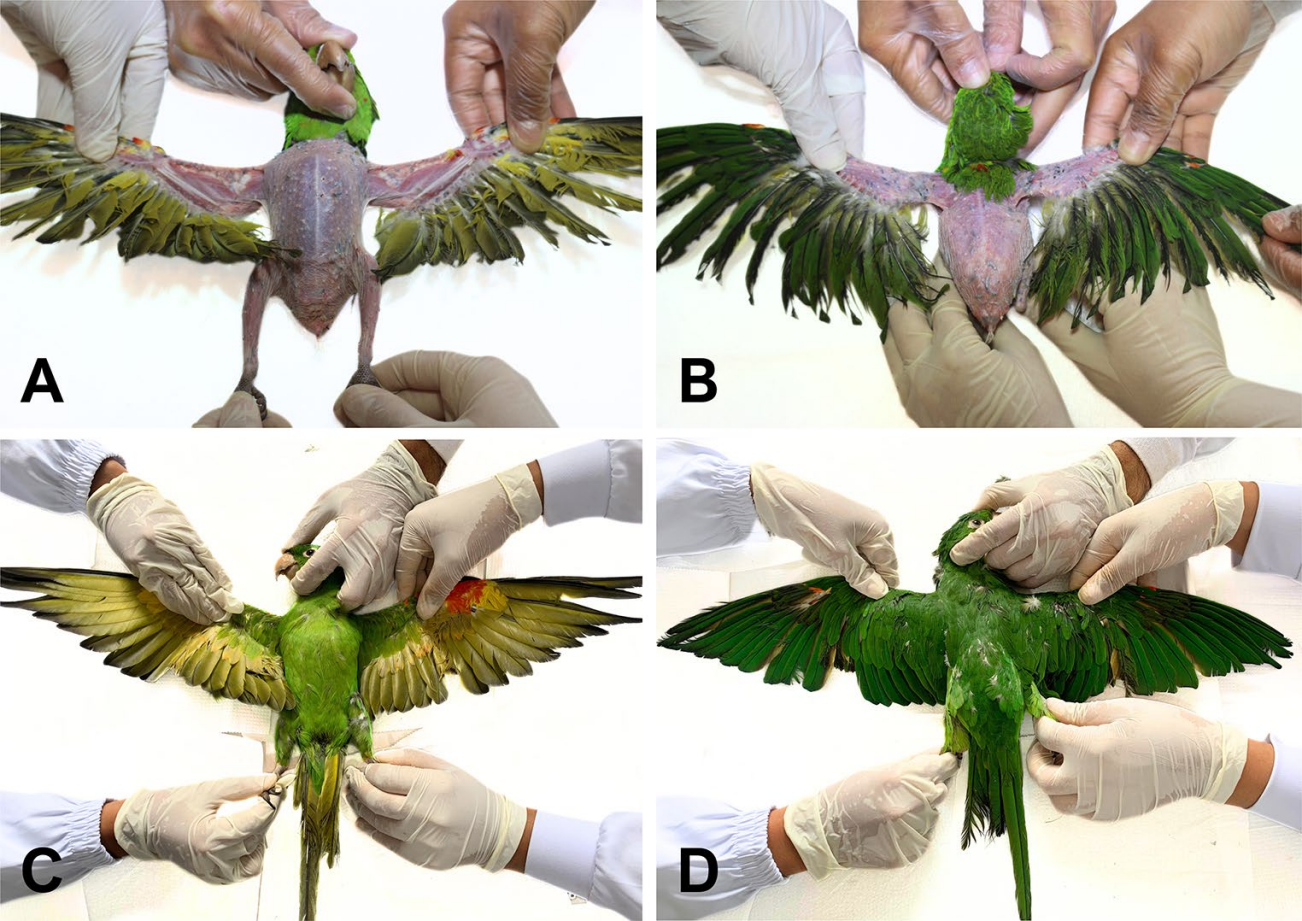
Examples of *Psittacara leucophthalmus* (white-eyed parakeet) specimens that were part of the study. (**A** and **B**) Individual with feather problems - score 0.75. (**C** and **D**) Individual with healthy feathers - score 10

Out of the 135 psittacines evaluated, 22 (16%) had a feather score lower than 10 (Fig. 2; Table 2), four (3%) exhibited overgrown beaks, and another three (2%) showed feather problems on the head (not included in the feather score). One individual was self-mutilating during the assessment. Some individuals had diarrhea, a dirty body or dirty cloacal feathers indicating lack of self-cleaning, and some were underweight. All animals had a body condition score of 3 (normal), except for five animals that scored 2 (thin). Linear regression (p-value = 0.6769), Spearman correlation (p-value = 0.6121), and Kendall correlation (p-value = 0.6556) tests revealed no association or correlation between the variables “feather score” and “stress index”.

### PCA: stress index and feather score

**Fig. 3:**
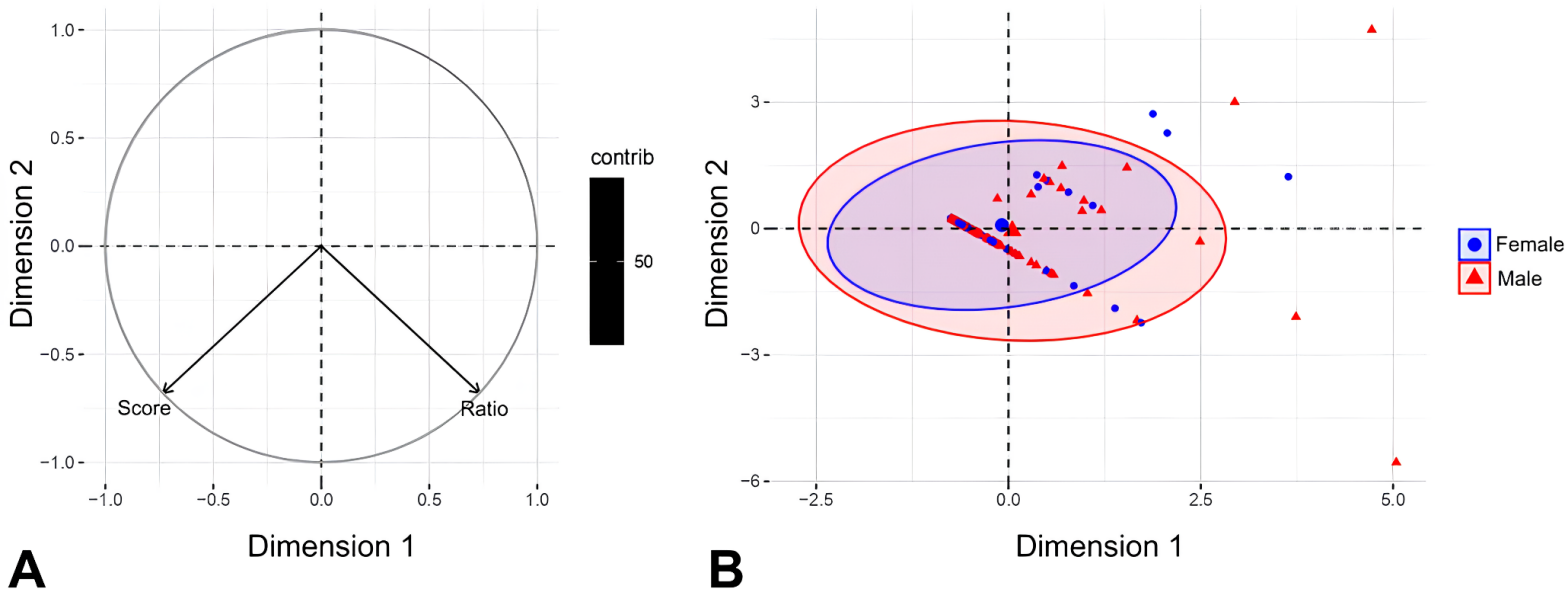
PCA (Principal Component Analysis) with data from all 135 psittacine birds included in the study. (**A**) Graph of the variables “feather score” and “stress index” (represented by the heterophil: lymphocyte ratio). (**B**) Graph of individuals separated by males and females

The PCA revealed no relationship between the variables “feather score” and “stress index” (Fig. 3-A), which confirmed the initial tests. Additionally, there was no statistical difference between these variables evaluated for males and females (Fig. 3-B).

### Molecular analyses

Three animals (3/135; 2.2%), all asymptomatic, were diagnosed as positive for the presence of *C. psittaci* (Table 1). These animals did not exhibit feather problems (feather score = 10) and did not present stress index above the overall average. They were two *Ara ararauna* (one female and one male) and one *Thectocercus acuticaudatus* (male). The latter died days after the biological sample collection, before the infection could be diagnosed and treated. The two *A. ararauna*, which had just arrived at the CETAS and were isolated from the other individuals in the flock, were treated with enrofloxacin and retested to confirm the elimination of the bacterium.

It was not possible to perform sequencing of the *C. psittaci* DNA fragments amplified due to the low DNA concentration after amplicon purification.

All birds, with or without clinical signs of feather loss, tested negative for *Circovirus* infection (Table 1).

## Discussion

More than half (85/135; 63%) of the psittacines allocated to CETAS-VDC during the period of this study belonged to the species *A. aestiva* and *E. cactorum*. These two psittacine species have been previously recorded as the most frequently received at a CETAS in a previous study carried out in the northeastern region of the country (Rio Grande do Norte, BR) [37]. Another study carried out in the microregion of Montes Claros (MG), on the border with Bahia, also report these two aforementioned species among the most frequently received psittacines at the CETAS in that region [38]. Research conducted in other regions of Brazil also identified the blue-fronted parrot as the most frequent psittacine bird at CETAS [39–41]. These data highlight the high demand for blue-fronted parrots (across Brazil) and cactus parakeets (in Bahia and nearby regions) as companion animals, as they are frequently encountered in seizures of trafficked wildlife.

The most efficient way to monitor animal stress is by measuring glucocorticoid metabolites in feces. However, this technique could not be employed in the present study due to its high cost. Although less commonly used, the H: L ratio can estimate animal stress and is more affordable. In general, a high level of stress was observed in the psittacines evaluated in this study. Vergneau-Grosset et al. [35], assessing the hematological profile of captive-born and healthy *Amazona amazonica* specimens, reported a stress index of 0.67, much lower than that reported here for this species and other psittacine birds in general. Schmidt et al. [36] provided hematological values for captive *Amazona vinacea* in a zoo, also resulting in a stress index lower than 0.7. Psittacine birds newly arrived at a CETAS can come from various sources: seized from trafficked shipments, rescued in urban areas, or voluntarily surrendered [42]. Birds taken from the wild or from long-term captivity may experience a temporary state of stress when introduced into a new environment like a CETAS [43]. For wild animals, this reaction can be triggered by the structure of aviaries, cages, or clinics, and by interaction with humans. Conversely, animals “domesticated” for many years may suffer from separation anxiety due to the disruption of their bond with the human who previously cared for them [44]. The findings of this study likely reflect the initial temporary stress upon the animals’ arrival at the CETAS, as most animals had biological samples collected shortly after arrival at the facility.

Birds positive for *C. psittaci* infection in this study did not show clinical signs, reinforcing that clinical evaluation alone should not be used as a diagnostic method for this pathogen. Several studies have also reported subclinical infection by *C. psittaci* in Brazilian psittacine bird. Raso et al. [13] found *A. aestiva* and *Anodorhynchus hyacinthinus* chicks positive for this bacterium in the Pantanal (Mato Grosso do Sul, BR) without any clinical signs. In a study in the southern region of Brazil (Paraná, BR), Ribas et al. [45] recorded the presence of *C. psittaci* in nests of *Amazona brasiliensis* without any clinical signs. Vasconcelos et al. [46], investigating *C. psittaci* in blue-and-yellow macaws from a CETAS in Rio de Janeiro (BR), observed that approximately 34% of the infected birds showed no compatible clinical alterations. Araujo et al. [47], studying psittacine birds from breeding facilities in Pará (BR), demonstrated that none of the animals diagnosed as positive for the bacterium exhibited clinical signs related to chlamydiosis. Santos et al. [48] carried out research in Bahia (BR) and recorded subclinical infection of *C. psittaci*, with a 3.4% positivity rate for native psittacine birds in captivity.

Only 2.2% (3/135) of the animals were found positive for *C. psittaci* infection in the present study. In 2018, an outbreak of chlamydiosis was first identified among the psittacine birds at the CETAS-VDC. Mirabal-Santos et al. [3] documented this outbreak, when 59 animals (*Amazona* spp.) were evaluated with 37% having tested positive for the pathogen. During 2021, another chlamydiosis outbreak was detected with over 80% of psittacine birds at the CETAS-VDC undergoing antibiotic treatment due to interactions between positive and negative animals in four out of five enclosures at the facility (data from Sacramento P., unpublished). Following these two outbreaks, alongside the findings of this study, a more stringent protocol for quarantine maintenance and screening for *C. psittaci* was implemented for all newly arrived psittacine birds at the facility. The low positivity rate observed here reflects the effectiveness of the new animal isolation protocol implemented at this CETAS since 2021, considering that not carrying out detection tests during this period could have revealed a higher number of positive animals among the resident population in this research.

The results found here for *C. psittaci* detection validate the importance of keeping newly arrived animals in quarantine, as indicated by Raso [8]. If the asymptomatic positive blue-and-yellow macaws had been placed directly into the same enclosure as other macaws at the CETAS-VDC, an outbreak could have been triggered leading to: (i) public expenditure on medication; (ii) overload of the institution’s professional staff; (iii) risk to the caregivers; (iv) morbidity and mortality of many animals; and (v) if all animals remained asymptomatic and were released without undergoing specific health assessments, the bacterium could have been introduced into the wild and spread to other species, including humans, since the genotype of the bacterium that infects psittacine birds has previously caused disease and fatalities in humans [5, 49].

The epidemiological assessment of *C. psittaci* infection carried out here sheds light on the importance of strict protocols to prevent pathogen dissemination in large facilities such as CETAS, zoos, and breeding centers. The quarantine methods employed here are still not ideal due to the associated high costs. Enhancements to the mentioned quarantine procedures would significantly exceed the budget of most Brazilian CETAS, such as (i) high-level disinfection of enclosures housing infected animals, (ii) complete disinfection of clothing worn by handlers in contact with quarantined animals, (iii) dedicated handlers exclusively for quarantined animals, and (iv) all examinations required by Normative Instruction (IN) IBAMA No. 05/2021.

Although the protocols employed here do not achieve the ideal of a perfect quarantine, through quarantine coupled with diagnostic testing, it was possible to prevent infected animals from transmitting the bacterium to others. IN IBAMA No. 05/2021 mandates testing for 15 avian pathogens, and due to financial constraints, some criteria need to be established for selecting which pathogens to assess for each species. In this context, some authors propose the concept of risk-based quarantine. This method relies on the animal’s history (e.g., origin and duration in captivity) and the risk it poses to animals at the receiving institution. It evaluates dangerous transmissible pathogens for the institution and, if none are identified, there is no need for risk mitigation (standard long-term quarantine can be eliminated) [50, 51]. McLean et al. [50] assessed morbidity and mortality in birds at Disney’s Animal Kingdom in Florida (USA) and found that birds subjected to risk-based quarantine had lower morbidity and mortality rates compared to those under standard quarantine processes. Given that *C. psittaci* is among the highest-risk pathogens for psittacines and has been reported circulating among them in Bahia [3, 48], the investigation of this pathogen as a risk factor for psittacines entering a CETAS in this state is of paramount importance.

Psittacine circovirus (BFDV) infection is common in psittacines worldwide and it has been detected in Brazil in the states of Rio de Janeiro, Rio Grande do Norte, Minas Gerais, and São Paulo [4, 19, 52, 53]. Therefore, it is necessary to implement epidemiological surveillance in other states in the country to investigate the presence of this pathogen and assess the potential inclusion of this diagnostic test in risk-based quarantine protocols for psittacines. This analysis becomes even more relevant in facilities that house birds exhibiting clinical signs indicative of infection, as observed in the animals included in this study (abnormal feathering and irregular beak growth).

This is the first epidemiological study for PBFD carried out in the state of Bahia, Brazil. There are six published studies reporting *Circovirus* diagnosis in psittacines in Brazil. Three of these studies were epidemiological investigations involving psittacines from the Brazilian states of Minas Gerais, São Paulo, Rio de Janeiro, Rio Grande do Sul, Paraná, and Mato Grosso do Sul; one study reported no positives [54], whereas two studies reported positivity rates of 6% [4] and 34% [19], including native species among the infected birds (*A. aestiva*, *A. amazonica*, *A. ararauna*, and *Triclaria malachitacea*). The other three studies are case reports of infected exotic animals: Batista et al. [52] evaluated *Melopsittacus undulatus* in Rio Grande do Norte, Ecco et al. [53] identified the virus in *Psittacula krameri* in Minas Gerais, and Werther et al. [55] diagnosed Beak and Feather Disease in *Cacatua alba* in the state of São Paulo.

Epidemiological studies like the one conducted here are important for assessing the spread of new pathogens among native animals. Early identification of viral infections, such as PBFD, allows for control measures to be implemented, preventing the spread of the pathogen that could lead to outbreaks, epidemics, or pandemics. Simply diagnosing the infection early can break a chain that could cause (i) ecological damage by transmitting to other wild psittacines, (ii) economic losses by spreading to pet or poultry birds, as the chicken anemia virus is phylogenetically close to BFDV in the same family Circoviridae (ICTV, 2011), and (iii) human public health risks, as animal-origin viruses can jump to humans, as seen, for example, in the emergence of SARS-CoV-2 [42, 56].

The non-identification of PBFD positives in psittacines with abnormal feathering may indicate that such conditions stem from behavioral disorders, such as psychogenic feather plucking. Atypical behaviors are common in captive animals due to stressful factors they are subjected to [57]. Therefore, the suggestion that feathering changes are linked to psychological rather than pathogenic infection is not unexpected, as previously described in the literature [58–60]. The animals evaluated here exhibited high stress levels, which made them susceptible to behavioral changes.

Another possibility considered for psittacines with feathering abnormalities observed in this study is infection by other pathogens, such as *Polyomavirus*. Studies involving the diagnosis of *Circovirus* commonly include tests for *Polyomavirus* as well. This is due to similar signs of feather loss and immunosuppression that can complicate the clinical diagnosis of both diseases [12, 17, 19]. Future research should expand the number of pathogens evaluated in psittacines with feathering issues to rule out all potential infections before releasing the animals.

Despite the lack of PBFD positive-tested psittacines, we suggest that animals showing symptoms consistent with the infection undergo diagnostic testing, as mandated by the IBAMA Normative Instruction No. 05/2021 governing CETAS operations [14]. This detection aims to make the best decision for animal welfare and the safety of its environment, whether it be (i) preventing the spread of the pathogen in case of a positive-tested animal or (ii) implementing strategies to reduce captivity-related stress, as feather changes in negative-tested animals are likely due to psychogenic feather plucking. An example of such a strategy is environmental enrichment, a tool that provides stimuli to increase natural species-specific behaviors and has been used in the treatment of psychogenic feather plucking in psittacines [60]. The team at the CETAS-VDC has been implementing this strategy, which has shown promising results. We also recommend epidemiological surveillance of this pathogen in the state of Bahia, given that positive cases have already been diagnosed in the northeastern region [52].

## Conclusion

*A. aestiva* and *E. cactorum* were the most frequent species at the CETAS in Vitória da Conquista, Bahia, during the study period. The animals received at the CETAS exhibited high levels of stress. Despite the low occurrence due to preventive measures, the results obtained here for *C. psittaci* associated with the infection history indicate a high risk of infection. This was the first epidemiological study of *Circovirus* (BFDV) in psittacine birds in the state of Bahia and revealed a low risk of infection by this pathogen in the region, given the lack of identification of positive animals. Therefore, we recommend that facilities with fewer resources prioritize periodic investigation for *C. psittaci*. In the present study, it was not possible to retest negative animals. However, we recommend that this measure be adopted in projects with higher funding, as the release of *C. psittaci* in feces is intermittent. Therefore, conducting multiple tests on negative animals with suspected infections is a safer protocol for animal health. Early diagnosis of pathogens in psittacine birds, as well as quarantine, is beneficial for wildlife and human health and is essential for animals under stress such as those housed in CETAS.

## References

[1] Hernandez EFT, Carvalho MS (2006) O tráfico de animais silvestres no Estado do Paraná. Acta Scientiarum Hum Social Sci 28:257–266

[2] Pavlin BI, Schloegel LM, Daszak P (2009) Risk of importing zoonotic diseases through wildlife trade, united States. Emerg Infect Dis 15:1721–1726. 10.3201/eid1511.090467 19891857 10.3201/eid1511.090467 PMC2857234

[3] Mirabal-Santos B, Antonio ES, Pereira DC, Dourado ATTS, Silva MB, Fraga RE, Tomazi L (2023) Determining the prevalence of avian chlamydiosis in wild Amazona species from Brazil using molecular testing and clinical signs. J Avian Med Surg 37:32–40. 10.1647/21-00075 10.1647/21-00075 37358200

[4] Araújo AV, Andery DA, Ferreira FC Jr, Ortiz MC, Marques MVR, Marin SY, Vilela DAR, Resende JS, Resende M, Donatti RV, Martins NRS (2015) Molecular diagnosis of beak and feather disease in native Brazilian psittacines. Brazilian J Poult Sci 17:451–458. 10.1590/1516-635x1704451-458

[5] Gaede W, Reckling KF, Dresenkamp B, Kenklies S, Schubert E, Noack U, Irmscher HM, Ludwig C, Hotzel H, Sachse K (2008) Chlamydophila psittaci infections in humans during an outbreak of psittacosis from poultry in Germany. Zoonoses Public Health 55:184–188. 10.1111/j.18632378.2008.01108.x 18387139

[6] Raso T, de Godoy F, Milanelo SN, Souza L, Matuschima CAI, Araújo ER, Pinto JP AA (2004) An outbreak of chlamydiosis in captive blue-fronted Amazon parrots (Amazona aestiva) in Brazil. J Zoo Wildl Med 35:94–96. 10.1638/02-090 15193081

[7] To KKW, Tse H, Chan W-M, Choi GKY, Zhang AJX, Sridhar S, Wong SCY, Chan JFW, Chan ASF, Woo PCY, Lau SKP, Lo JYC, Chan KH, Cheng VCC, Yuen KY (2014) A novel Psittacine adenovirus identified during an outbreak of avian chlamydiosis and human psittacosis: zoonosis associated with virus-bacterium coinfection in birds. PLoS Negl Trop Dis 8:e3318. 10.1371/journal.pntd.0003318 10.1371/journal.pntd.0003318 PMC4256287 25474263

[8] Raso TF (2014) Clamidiose: novas abordagens diagnósticas e terapêuticas. In: Cubas ZS, Silva JCR, Catão-Dias JL (eds) Tratado de animais Selvagens: medicina veterinária, 2nd edn. Roca, São Paulo, pp 2846–2872

[9] Raidal SR (2012) Avian circovirus and polyomavirus diseases. In: Miller RE, Fowler M (eds) Fowler’s zoo and wild animal medicine: current therapy. Saunders, Saint Louis, pp 297–303

[10] Phalen DN (2010) Implications of viruses in clinical disorders. In: Harrison GJ, Lightfoot TL (eds) Clinical avian medicine. Spix Publishing, Palm Beach, pp 573–586

[11] Schmidt RE, Reavill DR, Phalen DN (2003) Pathology of pet and aviary birds, 2nd edn. Wiley Blackwell, Ames

[12] Bert E, Tomassone L, Peccati C, Navarrete MG, Sola SC (2005) Detection of beak and feather disease virus (BFDV) and avian polyomavirus (APV) DNA in Psittacine birds in Italy. J Veterinary Med Ser B 52:64–68. 10.1111/j.1439-0450.2005.00823.x 15752264

[13] Raso T, de Seixas F, Guedes GHF, Pinto NMR AA (2006) Chlamydophila psittaci in free-living Blue-fronted Amazon parrots (Amazona aestiva) and hyacinth macaws (Anodorhynchus hyacinthinus) in the Pantanal of Mato Grosso do Sul, Brazil. Vet Microbiol 117:235–241. 10.1016/j.vetmic.2006.06.025 10.1016/j.vetmic.2006.06.025 16893616

[14] Brasil (2021) Instrução Normativa IBAMA N. 05 De 13 De Maio De 2021 Diário Oficial Da União 98(se–o 1):187–213

[15] Santos MC, Gomes DM, Lima MR, Santos MVB, Santos UG, Cerqueira RB, Macêdo JTSA, Pedroso PMO (2021) Quantitative study of wild animals received at the wild animals triage centers (CETAS) in Bahia and identification of trafficking routes. Pesquisa Veterinária Brasileira 41. 10.1590/1678–5150-PVB–6942

[16] Raso TF, Carrasco AOT, Silva JCR, Marvulo MFV, Pinto AA (2010) Seroprevalence of antibodies to Chlamydophila psittaci in zoo workers in Brazil. Zoonoses Public Health 57:411–416. 10.1111/j.1863–2378.2009.01237.x 19538456

[17] Kessler S, Heenemann K, Krause T, Twietmeyer S, Fuchs J, Lierz M, Corman VM, Vahlenkamp TM, Rubbenstroth D (2020) Monitoring of free-ranging and captive Psittacula populations in Western Europe for avian Bornaviruses, circoviruses and polyomaviruses. Avian Pathol 49:119–130. 10.1080/03079457.2019.1681359 10.1080/03079457.2019.1681359 31617746

[18] Sutherland M, Sarker S, Vaz PK, Legione AR, Devlin JM, Macwhirter PL, Whiteley PL, Raidal SR (2019) Disease surveillance in wild Victorian cacatuids reveals co-infection with multiple agents and detection of novel avian viruses. Vet Microbiol 235:257–264. 10.1016/j.vetmic.2019.07.012 31383310 10.1016/j.vetmic.2019.07.012

[19] Philadelpho NA, Chacón RD, Forero AJD, Guimarães MB, Astolfi-Ferreira CS, Ferreira AJP (2022) Detection of aves polyomavirus 1 (APyV) and beak and feather disease virus (BFDV) in exotic and native Brazilian Psittaciformes. Brazilian J Microbiol 53:1665–1673. 10.1007/s42770-022-00785–3 PMC9433492 35767215

[20] Harkins GW, Martin DP, Christoffels A, Varsani A (2014) Towards inferring the global movement of beak and feather disease virus. Virology 450–451:24–33. 10.1016/j.virol.2013.11.033 10.1016/j.virol.2013.11.033 24503064

[21] Campbell TW (2015) Hematologia de aves. In: Thrall MA, Weiser G, Allison RW, Campbell TW (eds) Hematologia e bioquímica clínica veterinária, 2nd edn. ROCA, São Paulo, pp 507–593

[22] Caldas EM (2000) Propedêutica clínica, 3rd edn. EDUFBA, Salvador

[23] Grespan A, Raso TF (2014) Psittaciformes (Araras, Papagaios, periquitos, Calopsitas e Cacatuas). In: Cubas ZS, Silva JCR, Catão-Dias JL (eds) Tratado de animais Selvagens: medicina veterinária, 2nd edn. Roca, São Paulo, pp 1172–1258

[24] Meehan CL, Millam JR, Mench JA (2003) Foraging opportunity and increased physical complexity both prevent and reduce psychogenic feather picking by young Amazon parrots. Appl Anim Behav Sci 80:71–85. 10.1016/S0168–1591(02)00192–2

[25] Fridolfsson A-K, Ellegren H (1999) A simple and universal method for molecular sexing of non-ratite birds. J Avian Biol 30:116–121. 10.2307/3677252

[26] Antonio ES, Batista PS, Teixeira SB, Barros JS, Gobbo GD, Fraga RE, Tomazi L (2021) Levantamento Da proporção sexual Em Ara chloropterus Gray, 1859 Em CETAS Da Bahia, Brasil. Revista Brasileira De Ciência Veterinária 28:218–224. 10.4322/rbcv.2021.056

[27] Fan HH, Kleven SH, Jackwood MW (1995) Application of polymerase chain reaction with arbitrary primers to strain identification of Mycoplasma gallisepticum. Avian Dis 39:729–735. 10.2307/1592409 8719206

[28] Hewinson RG, Griffiths PC, Bevan BJ, Kirwan SES, Field ME, Woodward MJ, Dawson M (1997) Detection of Chlamydia psittaci DNA in avian clinical samples by polymerase chain reaction. Vet Microbiol 54:155–166. 10.1016/S0378–1135(96)01268–0 9057259

[29] Flammer K (1994) Antimicrobial therapy. In: Ritchie BW, Harrison GJ, Harrison LR (eds) Avian medicine: principles and application. Wingers Publishing, Lake Worth, pp 434–456

[30] Fowler ME, Cubas ZS (2001) Biology, medicine, and surgery of South American wild animals. Wiley, Ames

[31] Green MR, Sambrook J (2017) Isolation of high-molecular-weight DNA using organic solvents. Cold Spring Harb Protoc 356–359. 10.1101/pdb.prot093450 10.1101/pdb.prot093450 28373491

[32] Ypelaar I, Bassami MR, Wilcox GE, Raidal SR (1999) A universal polymerase chain reaction for the detection of Psittacine beak and feather disease virus. Vet Microbiol 68:141–148. 10.1016/s0378–1135(99)00070-x 10501171

[33] Kassambara A (2017) Factoextra: extract and visualize the results of multivariate data analyses. R Package Version 1:0

[34] R Core Team (2022) R: A language and environment for statistical computing

[35] Vergneau-Grosset C, Polley T, Holt DC, Vernau W, Paul-Murphy J (2016) Hematologic, plasma biochemical, and lipid panel reference intervals in Orange-winged Amazon parrots (Amazona amazonica). J Avian Med Surg 30:335–344. 10.1647/2015–129 28107066

[36] Schmidt EM, dos Lange S, Ribas RR, Daciuk JM, Montiani-Ferreira BM, Paulillo F AC (2009) Hematology of the red-capped Parrot (Pionopsitta pileata) and vinaceous Amazon Parrot (Amazona vinacea) in captivity. J Zoo Wildl Med 40:15–17. 10.1638/2007–0054.1 19368236

[37] Oliveira ES, Torres D, de Alves F (2020) RR da N Wild animals seized in a state in Northeast Brazil: Where do they come from and where do they go? Environ Dev Sustain 22:2343–2363. 10.1007/s10668-018-0294–9

[38] Franco MR, Câmara FM, Rocha DCC, Souza RM, Oliveira NJF (2012) Animais silvestres Apreendidos no período de 2002 a 2007 Na macrorregião de Montes Claros, Minas Gerais. Enciclopédia Biosfera 8:1007–1018

[39] Freitas ACP, Oviedo-Pastrana ME, Vilela DA da, Pereira R, Loureiro PLL, de OC L, Haddad JPA, Martins NR da, de Soares S (2015) M Diagnóstico de animais ilegais recebidos no centro de triagem de animais silvestres de Belo Horizonte, Estado de Minas Gerais, no ano de 2011. Ciencia Rural 45:163–170. 10.1590/0103–8478cr20131212

[40] Moura SG, Pessoa FB, Oliveira FF, Lustosa AHM, Soares CB (2012) Animais silvestres Recebidos Pelo Centro de triagem do IBAMA no Piauí no Ano de 2011. Enciclopédia Biosfera 8:1748–1762

[41] Souza T, de O, Vilela DA, da Câmara R O (2014) Pressões sobre a avifauna Brasileira: Aves Recebidas Pelo CETAS/IBAMA, Belo Horizonte, Minas Gerais. Ornithologia 7:1–11

[42] Destro GFG, Pimentel TL, Sabaini RM, Borges RC, Barreto R (2012) Efforts to combat wild animals trafficking in Brazil. In: LAMEED GA (ed) Biodiversity enrichment in a diverse world. InTechOpen, Londres, pp 421–436

[43] Dickens MJ, Earle KA, Romero LM (2009) Initial transference of wild birds to captivity alters stress physiology. Gen Comp Endocrinol 160:76–83. 10.1016/j.ygcen.2008.10.023 19026651 10.1016/j.ygcen.2008.10.023

[44] Gaskins LA, Hungerford L (2014) Nonmedical factors associated with feather picking in pet Psittacine birds. J Avian Med Surg 28:109–117 25115039 10.1647/2012-073R

[45] Ribas JM, Sipinski EAB, Serafini PP, Ferreira VL, Raso T, de Pinto F AA (2014) Chlamydophila psittaci assessment in threatened red-tailed Amazon (Amazona brasiliensis) parrots in Paraná. Brazil Ornithologia 6:144–147

[46] Vasconcelos TCB, Nogueira DM, Pereira VL, de Nascimento A, Bruno ER SF (2016) Chlamydia psittaci in captive blue-and-gold macaws (Ara ararauna) in a triage center of wild animals in Brazil. Revista Brasileira De Ciência Veterinária 23:37–41. 10.4322/rbcv.2016.027

[47] Araujo SAA, Pereira WLA, Silva SP, Cardoso JF, Silva-Filho E, Bernal MKM, Mendes FF, Nunes MRT (2019) Clinical and molecular diagnosis of Chlamydophila in captive parrots in Pará State, Brazil. Semin Cienc Agrar 40:2603–2612. 10.5433/1679–0359.2019v40n6p2603

[48] Santos F, Leal DC, Raso TF, Souza BMPS, Cunha RM, Martinez VHR, Barrouin-Melo SM, Franke CR (2014) Risk factors associated with Chlamydia psittaci infection in Psittacine birds. J Med Microbiol 63:458–463. 10.1099/jmm.0.060632–0 24430249

[49] Martins ARB, Barkokébas B, Lago EMG, Vasconcelos BM, Cruz FM, Zlatar T, França TCM, Pedrosa LR (2018) Occupational safety and health for workers managing wild animals. In: Arezes PM, Baptista JS, Barroso MP, Carneiro P, Cordeiro P, Costa N, Melo RB, Miguel AS, Perestrelo G (eds) Occupational safety and hygiene VI, 1st edn. CRC, Boca Raton, pp 143–148

[50] McLean KM, Schook MW, Pye GW (2020) Comparison between standard zoo quarantine practices and risk-based management of animal transfers: A retrospective analysis of avian acquisition morbidity and mortality (2013–2018). J Zoo Wildl Med 51:1017–1020. 10.1638/2017–0239 33480584

[51] Ramsay EC, Steeil J, Cross T, Souza M (2013) A risk-based quarantine program: an alternative to one size fits all. In: American Association of Zoo Veterinarians Conference 2013

[52] Batista AIV, Pereira AWdaS, Prazeres-Júnior FR, Gurgel JV, de O, Pereira LMF, Araújo BVS, Moreira A, de Medeiros C, Freitas NO CIA (2021) Generalized aptheria and automutilization of members in Budgegarigar (Melopsittacus undulatus SHAW, 1805) presenting circovirosis in the Northeast of Brazil. Res Soc Dev 10. 10.33448/rsd-v10i13.20864

[53] Ecco R, Silva LMN, Lacerda M, dos SC, Moraes MV dos, de S, de Oliveira LB, Santos WH, de Rizotto M, Saraiva LS, Bueno LHG, Dorlass LM, Durigon EG, Spilki EL, Ferreira FR (2022) HL First detection of Psittacid alphaherpesvirus 5 and coinfection with beak and feather disease virus in naturally infected captive ringneck parakeets (Psittacula krameri) in Brazil. Arch Virol 167:2319–2324. 10.1007/s00705-022-05566–9 35962822

[54] Vaz FF, Sipinski EAB, Seixas GHF, Prestes NP, Martinez J, Raso TF (2021) Molecular survey of pathogens in wild Amazon Parrot nestlings: implications for conservation. Divers (Basel) 13:272. 10.3390/d13060272

[55] Werther K, Raso TF, Durigon EL, Latimer KS, Campagnoli RP (1999) Doença do Bico e Das Penas no Brasil: Relato de Caso. Brasilian J Poult Sci 1:85–88

[56] Hassanin A, Grandcolas P, Veron G (2020) Covid–19: natural or anthropic origin? Mammalia 85:1–7

[57] Morgan KN, Tromborg CT (2007) Sources of stress in captivity. Appl Anim Behav Sci 102:262–302. 10.1016/j.applanim.2006.05.032

[58] Ebisawa K, Nakayama S, Pai C, Kinoshita R, Koie H (2021) Prevalence and risk factors for feather-damaging behavior in Psittacine birds: analysis of a Japanese nationwide survey. PLoS ONE 16. 10.1371/journal.pone.0254610 10.1371/journal.pone.0254610 PMC8279392 34260621

[59] Jayson SL, Williams DL, Wood JLN (2014) Prevalence and risk factors of feather plucking in African grey parrots (Psittacus erithacus erithacus and Psittacus erithacus timneh) and cockatoos (Cacatua spp). J Exot Pet Med 23:250–257. 10.1053/j.jepm.2014.06.012

[60] Telles LF, Malm C, Melo MM, Vilela DA, da Lago R, Silva LA, Martins MX (2015) NR da S Arrancamento de penas psicogênico em maritacas: haloperidol e enriquecimento ambiental. Ciencia Rural 45:1099–1106. 10.1590/0103–8478cr20140318

